# Motives for Filing a False Allegation of Rape

**DOI:** 10.1007/s10508-017-0951-3

**Published:** 2017-02-17

**Authors:** André W. E. A. De Zutter, Robert Horselenberg, Peter J. van Koppen

**Affiliations:** 1 Department of Criminal Law and Criminology, Faculty of Law, VU University Amsterdam, De Boelelaan 1105, 1081 HV Amsterdam, The Netherlands; 2 Department of Criminal Law and Criminology, Faculty of Law, Maastricht University, P.O. Box 616, 6200 MD Maastricht, The Netherlands

**Keywords:** Rape, False allegations, Police records, Motives of complainants

## Abstract

The list of motives by Kanin (1994) is the most cited list of motives to file a false allegation of rape. Kanin posited that complainants file a false allegation out of revenge, to produce an alibi or to get sympathy. A new list of motives is proposed in which gain is the predominant factor. In the proposed list, complainants file a false allegation out of material gain, emotional gain, or a disturbed mental state. The list can be subdivided into eight different categories: material gain, alibi, revenge, sympathy, attention, a disturbed mental state, relabeling, or regret. To test the validity of the list, a sample of 57 proven false allegations were studied at and provided by the National Unit of the Dutch National Police (NU). The complete files were studied to ensure correct classification by the NU and to identify the motives of the complainants. The results support the overall validity of the list. Complainants were primarily motivated by emotional gain. Most false allegations were used to cover up other behavior such as adultery or skipping school. Some complainants, however, reported more than one motive. A large proportion, 20% of complainants, said that they did not know why they filed a false allegation. The results confirm the complexity of motivations for filing false allegations and the difficulties associated with archival studies. In conclusion, the list of Kanin is, based on the current results, valid but insufficient to explain all the different motives of complainants to file a false allegation.

## Introduction

### The Problem of False Allegations

False allegations of rape can cause problems for all parties involved. In Germany, for instance, a female teacher accused a male colleague of rape. Heidi K. claimed that Horst Arnold had raped her in 2002 in the biology classroom. Arnold was convicted of the crime and sentenced to 5-year imprisonment. He served the full sentence. He was denied parole because he continued to proclaim his innocence. He was acquitted in a retrial in 2011. Arnold died in Saarland, Germany, in 2012 due to heart failure. On September 13, 2013, Heidi K. was convicted for 5½ years imprisonment for deprivation of freedom due to a false allegation of rape (Friedrichsen, 2013; Sapa, 2013). The case illustrates that a false allegation can result in consequences not only for the falsely accused but also for the complainant.

In the U.S., a false complainant could also be prosecuted and convicted. We are, however, unaware of the actual prosecution and conviction rate of false complainants in the USA as well as the content of such convictions. A miscarriage of justice in the U.S., however, might be indicative of the fate of false complainants of rape in the U.S. On March 12, 2009, Marie was offered a plea deal after she retracted her allegation of rape, and confessed that the allegation was false The plea deal consisted of mental health counseling and supervised probation for a year, and a fine of 500 $. Marie accepted the plea deal, despite the fact that she was a victim of the serial rapist, Marc O’Leary. In 2011, Marie was exonerated, and her record was expunged (Armstrong & Miller, 2015). Besides the judicial consequences, a false complainant might suffer psychologically from the moral consequences of filing a false allegation. An illustration hereof is the notorious case of Gary Dotson. Kathleen Crowell Webb fabricated a rape after unprotected consensual sex with her boyfriend to obtain contraceptive medication. Coincidentally, Gary Dotson resembled Kathleen’s fabricated rapist and was convicted of rape (Webb & Chapian, 1985). Kathleen recanted her false allegation of rape out of remorse, but her initial allegation was so convincing that some scholars and the judge who reviewed the case did not believe her retraction (Taylor, 1987). Gary Dotson spent years in and out of prison as a consequence of the false allegation and it was not until the advent of DNA research that he was exonerated (Heath, 2009). Kathleen wrote a book with the self-explanatory title “Forgive Me” (Webb & Chapian, 1985). False allegations of rape are not a myth but are not ubiquitous either. Ferguson and Malouff (2016) found a rate of 5% confirmed false allegations in their meta-analysis on seven studies on the prevalence of false allegations.

### Motives for Filing a False Allegation

Women who have been raped often find criminal proceedings distressing. Sometimes, these criminal proceedings are referred to as secondary victimization (Bohmer & Blumberg, 1975; Doherty & Anderson, 1998; Latts & Geiselman, 1991). Why would an individual willfully and wittingly file a false allegation? In the case of Heidi K. and Arnold, it is assumed that rivalry was the ground for the false allegation. Heidi K. would have thought that her chances of getting a promotion would increase considerably if she could dispose of Arnold (Sapa, 2013). The false allegation was therefore used to gain something.

Kanin (1994) described three motives for filing a false allegation: alibi, revenge, and attention/sympathy seeking. Kanin based his list on a study he conducted in a police agency of a small metropolitan town with approximately 70,000 inhabitants in the U.S., from 1978 to 1987. Kanin collected all false allegations (*N* = 45) made to the police in that period. An allegation was considered false if the complainant retracted her allegation and admitted that she had filed a false allegation. The complainant had to state that she was not raped to fulfill his criterion of an allegation being false. The list by Kanin was a data-driven list. It means that the list was based on the verbalizations of the complainants during their recantation of the rape allegation.

The three motives reported by Kanin (1994) seem valid and anecdotal evidence for each motive exists. When a false rape allegation serves the alibi function, the allegation is used to cover up other behavior. In IJmuiden, the Netherlands, a woman used a false allegation of rape to cover up an adulterous affair. Her partner then caught the complainant in the act of adultery with an acquaintance. In an attempt to hide the adultery, she accused the acquaintance of raping her. Police investigation revealed that the allegation was false (ANP, 2010). In another case in the Netherlands, in Noordwijk, a 17-year-old girl used a false allegation as an explanation for being too late for her apprenticeship (Novum, 2010).

In revenge cases, according to Kanin (1994), the allegation is used to retaliate. On March 24, 1988, Inge V. filed an allegation of rape against the ex-lover of her mother, Ad Schagen (Korver, 1991b, 1991c). The alleged rapist supposedly used a lot of violence, ripping the clothes of Inge, hitting, strangulating, and tying her to the bed. Inge V. gave detailed descriptions of the alleged rapes to the police. Nobody believed in the innocence of Ad Schagen. It was not until his second lawyer studied the criminal file painstakingly that doubts concerning the truthfulness of the allegation arose. Inge V., for example, claimed to have been raped by Ad Schagen while her mother was playing tennis. The lawyer discovered that the mother of Inge V. did not play tennis at all. The lawyer made a list of all the discrepancies, inconsistencies, and contradictions and forwarded the list to the public prosecutor. The list led the public prosecutor to dismiss the case. In 1991, Inge V. confessed to the police that she had filed a false allegation. Later that year, she said in an interview with journalist Henny Korver in *De Telegraaf* newspaper that she had filed the allegation out of revenge. She hated the lovers of her mother and wanted to hurt Ad Schagen and her mother (Korver, 1991a). She was convicted of filing a false allegation of rape and was given a suspended sentence of 6 months and a fine of 100 Dutch guilders (±50 euro) (Korver, 1991d).

If the third kind of motives applies, when an allegation is used to attract sympathy or attention, the rape is usually disclosed to close friends or caregivers and involves unknown perpetrators. Kanin (1994) concluded that this motive for filing an allegation was the most socially harmless. Even if most false allegations that are done to attract attention involve unknown perpetrators, it certainly is not always the case. On March 18, 2009, at 11:28 p.m. a woman phoned the Dutch police that she had been abducted and raped in a car. She gave a detailed description of the car. On March 29, 2009, at 12.27 p.m., the same woman called the police again and told the police that she had been raped once more. The perpetrators managed to get hold of her when she opened the door of her home to walk her dogs. The perpetrators tied her to her chair, blindfolded her, and raped her. Later, she was tied to her bed and was repeatedly raped in a cruel manner. The alleged rapists had been pinching her, pulling her hair, and beating her. She called the police again on April 20, 2009, at 1:12 p.m. and filed a new allegation of rape later that day. Because of her detailed description of the car, the alleged rapists could promptly be identified. The men had alibis for one of the events, and the police investigation revealed that she had faked the rapes herself. Because of the sadomasochistic nature of the consensual sexual encounters the woman had, rape-consistent injuries were present. On July 26, 2011, she was convicted of repeatedly filing a false allegation of rape. She had filed the false allegations of rape to attract attention. Psychologists diagnosed her with a histrionic and borderline personality disorder (District Court Zutphen, 2011).

### The Shortcomings of the List

The three major categories of alibi, revenge, and sympathy or attention were also found by other researchers (McNamara, McDonald, & Lawrence, 2012). McNamara et al. studied 30 false allegations that were submitted by other law enforcement agencies to the Federal Bureau of Investigations’ (FBI) National Center for the Analysis of Violent Crime (NCAVC) during a 15-year period. The majority of false allegations were filed by women (*n* = 22). Thirteen women filed a false allegation of rape, while nine women filed nonsexual false allegations. The men in the sample all filed nonsexual false allegations. The nonsexual false allegations involved crimes such as stalking, threats, abduction, attempted murder, and extortion. It is impossible to draw any conclusion based on the study by McNamara et al. due to the small and atypical sample. Nevertheless, McNamara et al. reported the same motives as Kanin (1994), thus supporting the list to some extent.

The list, however, is not exhaustive. For example, the motive of gain is not included in the list. One could argue that revenge, an alibi, and attention or sympathy are also some form of gain. The difference, however, is that revenge, an alibi, and attention or sympathy are emotional gains while a motive like a promotion to a better job is more a material gain. McNamara et al. (2012) also reported that some complainants were motivated by profit, i.e., material gain. But there are still other motives for a complainant to file a false allegation. McNamara et al. reported one more motivator, mental illness. Sometimes complainants file false allegations of rape following sexual hallucinations (Balasubramaniam & Park, 2003). The complainants are convinced that they were raped and had no intention to file a false allegation. Their lack of intention makes it a special subgroup of complainants. In other cases, complainants file a false allegation as a consequence of pseudologia fantastica, i.e., pathologic lying (Dubois, 1987). Thus, complainants can file a false allegation because of a disturbed mental state.

A Dutch defense lawyer, Veraart (2006), described two other motives for filing a false allegation. Sometimes consensual sex is afterward presented by the complainant as rape to the police, because of its disappointing or shameful character. The relabeling, however, is not internalized as the complainant is still aware of the fact that she was not raped at all because the sexual encounter was consensual. If consensual sex afterward is, due to external pressure or influence, relabeled as rape, the complainant might not have desired the sexual encounter but did consent without any abuse of power or manipulation by the other party. The complainant, however, did not convey her lack of desire. Unwanted but consensual sex is common (Bay-Cheng & Eliseo-Arras, 2008; Erickson & Rapkin, 1991; O’Sullivan & Allgeier, 1998; Philips, 2000). In the study conducted by O’Sullivan and Allgeier, 26% of men and 50% of women reported at least one occasion in which they had engaged in unwanted, but consented, sexual activity in a 2-week period. The element of a not wanting, a lack of desire, is used to justify the false allegation of rape. But the complainant is still aware of the fact that she was not raped and consented to the sexual encounter. Lay people tend to associate rape with not wanting. De Zutter, Horselenberg, and van Koppen (2017) conducted a quasi-experiment in which they asked 35 women to fabricate rape and file a false allegation. They found that the fabricated stories of rape, the false allegations, resembled unwanted sex. Studies on fabricated rape have consistently shown that lay people tend to associate not wanting sex with rape (De Zutter, Horselenberg, & van Koppen, 2016; De Zutter et al., 2017). Thus, if a complainant recounts her unwanted consensual sexual encounter to friends and family, her social environment will react with the label of rape. Once the consensual sexual encounter is labeled rape by the environment, it creates a proverbial point of no return in the head of the complainant who decides to file a false allegation of rape at the police station instead of confronting her social environment with the assertion that their label is invalid (Veraart, 1997, 2006). Sometimes scholars have been said to engage in the process of relabeling consensual sexual encounters as rape. Sommers (1995) argued in her book “Who Stole Feminism?” that relabeling by scholars caused an inflation of the prevalence rates of rape reported by some scholars in the USA, because only one in four women who were labeled victims of rape by scientists in these studies believed that they were, in fact, raped.

If regret is the motive to file a false allegation, the complainant experiences negative feelings such as disgust, shame, and sorrow. The negative feelings are typically noticed by close friends or relatives who will ask about the source of the negative feelings. The sexual encounter may then be labeled as rape by others. The complainant may not have the courage to admit that she also played a vital role in the sexual encounter. The complainants are often persuaded by others to file a false allegation (Veraart, 1997).

In sum, there are several motives to file a false allegation: material gain, alibi, revenge, sympathy, attention, a disturbed mental state, relabeling, or regret. Gain is the underlying driving force of every form of motive with one exception: Complainants with sexual hallucinations have no interest in either emotional or material gain. Although most motives can be reduced to some form of gain, the underlying emotional states are so diverse that it makes sense to treat them as separate motives. As a consequence, we argue that the list proposed by Kanin (1994) is not adequate because it does not cover all the motives provided by complainants.

We propose an expanded list in which gain is the predominant factor. In the list, complainants file a false allegation out of material gain, emotional gain, or mental disturbance. The list can be subdivided into eight different categories: material gain, alibi, revenge, sympathy, attention, a disturbed mental state, relabeling, or regret. The aim of the current study was to test the validity of the list.

### Ground Truth

In studies in which the truthfulness of allegations of rape plays a role, it is important to establish ground truth. Ground truth is a term used to define what happened (DeAndrea, Tom Tong, Liang, Levine, & Walther, 2012; Horowitz, 2009; Iacono, 2008; Swets, 1988). The term is often used to define the accuracy of diagnostic systems, and the outcome of a test is compared to the ground truth (Swets, 1988). If we apply ground truth to false allegations of rape, it means that allegations classified as false are, in reality, false allegations of rape, while allegations classified as true are, in reality, true allegations of rape. In that sense, false negatives, true allegations in the sample of false allegations should be avoided as much as possible.

Researchers on allegations of rape have used different concepts to represent ground truth in their studies. Some researchers used the judicial outcome as a substitute for ground truth (Rassin & Van der Sleen, 2005). That is, however, not a correct representation of ground truth because sometimes guilty people are discharged, and sometimes innocent people get convicted, as in the case of Gary Dotson (Gross, Jacoby, Matheson, Montgomery, & Patil, 2005). Other researchers deemed all allegations of rape to be true unless they received the unfounded or no crime-label by police officers (Rumney, 2006). In that case, police officers decide whether an allegation of rape is false. Police officers, however, sometimes use the unfounded or no crime-label incorrectly. Police officers label allegations as unfounded in the case of marital rape or due to a variety of evidence problems, regardless of the ground truth (Gregory & Lees, 1996). A final approach is to take a retraction by the claimant as proof of a false allegation (Kanin, 1994). Sometimes claimants, though, retract their allegation due to police pressure (e.g., when they are not believed or told that there is no possibility to obtain a conviction) (Haket, 2007). In conclusion, it is not easy to obtain ground truth. Therefore, stringent criteria should be used in studies on allegations of rape to avoid false negatives.

## Method

### Subjects

The total sample of false allegations of rape that were studied to identify the motive for filing a false allegation consisted of 57 cases. Rape was defined consistent with Dutch law. It was, therefore, defined as the actual unlawful compelling of a person through physical force or duress to have sexual intercourse. Allegations of complainants under the age of 14 were excluded because in the Netherlands people under the age of 14 are lawfully unable to consent to any sexual activity. The study was limited to fabricated male rapists and fabricated female victims of rape. Thus, all false complainants were women, and over the age of 14. All false allegations of rape were drawn from the files of the National Unit of the Dutch National Police (NU). Permission to study the files and to gather data was granted by the Minister of Security and Justice of The Netherlands. We fulfilled all conditions as stated in the permission by the Minister. Thus, no demographic data were collected, all raw data were anonymized to protect the identity and secure confidentiality of all parties involved, and files were only identifiable through a number and were studied and coded at the headquarters of the NU in the Netherlands.

### Measures and Procedure

False allegations of rape were defined as deliberate fabrications of rape while the complainant was not raped. A case was added to the sample of false allegations if the complainant retracted the allegation and said that the allegation was, in fact, false and no rape whatsoever had occurred. Also, the alternative scenario had to be supported by corroborative and conclusive evidence. Thus, a retraction was the first and necessary step, the second step was a thorough investigation to proof that the allegation was false, and the third step was evidence of what truly happened which corroborated the retraction or confession of the complainant. For instance, a complainant said that she was raped in an alley. Forensic examination revealed vaginal seizures and the presence of semen. A male DNA profile could be obtained from the semen. The DNA profile did not match with any of the profiles in the Dutch police DNA database. The alley, however, was equipped with surveillance cameras. The police examined the footage and did not see the complainant. When the complainant was confronted with the footage, she retracted her allegation. She admitted that she had not been raped by a stranger but had had consensual sex with her boyfriend. The DNA profile did match with the DNA sample provided by her boyfriend. She admitted that she had invented the rape out of fear of her father. She told the police that her father was a racist and did not approve of her relationship with her boyfriend of foreign descent. In conclusion, our definition conformed to the guidelines of the Uniform Crime Reporting (UCR) Program of the Federal Bureau of Investigations (FBI) and the International Association of Chiefs of Police to unfound allegations of rape as well as the definition used by Ferguson and Malouff (2016) in their meta-analysis. The UCR guidelines of the FBI state that a law enforcement agency in the U.S. has to establish through investigation that the reported rape did not occur in order to deem a complaint unfounded (FBI, 2010; UCR, 2004). Our definition was, however, more strict to establish ground truth as much as possible.

We found the cases using the Violent Crime Linkage System (ViCLAS) of the NU. ViCLAS is a software program developed by the Royal Canadian Mounted Police (Aldred, 2007). The database is used to analyze violent crimes to detect patterns and catch serial offenders. The Dutch police try to enter all murders with a sexual motive and sexual offenses in the Netherlands into ViCLAS. On average, NU officers entered 494 sexually motivated offenses in ViCLAS per year. Out of the 494 cases entered in ViCLAS, 195 were rape cases.^1^

Since 2002, all entries were made by trained NU officers on the basis of a structured questionnaire. Law enforcement agencies across the Netherlands send criminal files of murders and sexual offenses to the NU. The motive for filing a false allegation is not part of the questionnaire. As a consequence, we had to study the original case files to identify the motive for filing a false allegation. To establish the ground truth, we studied the complete case files. ViCLAS was only used to enter queries and identify potentially relevant files.

All 91 allegations that were classified as false in ViCLAS from April 1997 until August 2011 were studied. Twenty of these files were incomplete. Thus, it was not possible to establish ground truth in these cases. Additional information was sought either through a national police search engine (called BlueView) or from the local police district. Additional information was obtained from nine files. Seven of these allegations met the criteria of the definition of a false allegation of rape used in the current study, but two did not. The current sample was part of a larger study on the differences between true and false allegations. Therefore, true allegations of rape were also studied. One false allegation that was misclassified as a true allegation by the NU was added to the sample of false allegations and was included in the current study.

In cases in which a complainant retracted her allegation of rape, the Dutch police protocol for vice cases prescribes a few steps that the police officers are obliged to follow. First, an interrogation of the complainant as a suspect has to take place. Second, the complainant has to explain why she retracted her allegation to prevent false retractions. Third, the police officer has to ask why she filed a false allegation. Fourth, and finally, the police officers have to document the interrogation and enter it into the criminal file. The verbalizations of the motives for filing the false allegation by the complainants were collected by the first author, which is a limitation because we were unable to calculate inter-coder reliability. Consecutively, the individual motives were classified as motivated by material gain, emotional gain, or a mental disturbance. Finally, the motivations were classified as motivated by material gain, alibi, revenge, sympathy, attention, a disturbed mental state, relabeling, or regret.

## Results

Complainants reported several different motives for filing a false allegation. In four cases, complainants reported more than one motive. Gain was the predominant factor in the current sample. We found no false allegation because of a disturbed mental state on the part of the complainant, such as sexual hallucinations. The majority of complainants were motivated by emotional gain (*n* = 35; see Table 1). One complainant was motivated by material gain (see Table 1). It was, however, not the only motivation (see Table 1). The complainant received money from Victim Care but was also motivated to file a false allegation to receive attention from friends, family, and Victim Care. Victim Care in the Netherlands is a government-funded organized interest group for victims. Among other things, they offer, free of charge, emotional support to victims. But Victim Care in the Netherlands can also award damages to victims of sexual or physical violence.

**Table 1:** Number and proportion of motives per category (*N* = 58) and subcategory (*N* = 61) of all false complainants (*N* = 57)

Category	*n*	%	Subcategory	*n*	%
Material gain	1	1.72	Material gain	1	1.64
Emotional gain	35	60.35	Attention	9	14.75
			Revenge	5	7.94
			Sympathy	3	4.92
			Alibi	14	22.95
			Mental disorder	2	3.28
			Relabeling	2	3.28
			Regret	3	4.92
Disturbed mental state	0	0.00	Disturbed mental state	0	0.00
I don’t know	12	20.69	I don’t know	12	19.67
Unknown	10	17.54	Unknown	10	16.39

*n* = the number of complainants that used the specific motivation; some complainants used more than one motive; therefore, the sum is more than 57; % = the proportion of the times the specific motivation is used in comparison with the sum of all motivations

The most frequently reported motivation to file a false allegation of rape was the so-called alibi subcategory (*n* = 14; see Table 1). These complainants used the false allegation of rape to cover up other behavior. The false allegation, for example, was used to cover up adultery, lateness, or skipping school. Nine complainants tried to gain attention by filing a false allegation. Five complainants stated that they filed a false allegation to take revenge on someone. Some complainants sought revenge because they felt betrayed after consensual sex because the other party only wanted a one-night stand (*n* = 3; see Table 1). One complainant filed a false allegation because her parents requested her to file a false allegation of rape. They wanted to take revenge on the daughter’s ex-boyfriend. It had been an abusive relationship according to the complainant’s parents. One complainant committed adultery with the falsely accused, regretted it, was afraid that the liaison would be discovered by her lover and blamed the accused for the trouble in which she found herself.

Some complainants sought sympathy by filing a false allegation (*n* = 3; see Table 1). Regret was the factor for another three complainants. Two complainants felt ashamed after they willingly participated in group sex and one complainant was ashamed after consensual sex. Two complainants relabeled their consensual sexual encounter as rape and consequently filed a false allegation of rape. For two complainants, psychopathology was the motivation to file a false allegation. One complainant, a girl with autism, thought she could make the park a safe place again by filing a false allegation. The other complainant was a woman with a histrionic and borderline personality disorder who stated that she was compelled to file a false allegation of rape. One complainant was driven by material gain and sought financial compensation from Victim Care (see Table 1).

Two categories had to be added to the proposed list. On the one hand, the “I don’t know” category was added, since some complainants stated that they did not know why they had filed a false allegation. It was considered as an additional category because these complainants insisted that they did not know, although the interrogating police officers in these cases pressured the complainants to provide a reason for filing a false allegation. The false complainants were suspects of a crime and were interrogated as such. In the Netherlands in suspect interrogations, some pressure is considered lawful. On the other hand, the “Unknown” category was added, since sometimes police officers did not ask the complainants why they had filed a false allegation or did not document the answer to that question. Twelve complainants stated that they did not know why they had filed a false allegation. In 10 cases, police officers did not ask or did not document what the motive for filing a false allegation was. Contrary to the mandatory instructions in the Dutch police protocol for vice cases, there was no information on the motive in the criminal files we studied.

## Discussion

Emotional gain was the predominant motivation for filing a false allegation. The majority, 60% of all complainants, had an emotional motive. Material gain was for one complainant one of the motives for filing a false allegation. Almost one in four complainants used the false allegation to cover up other behavior. False allegations were used to cover up adultery, lateness, consensual sex, and skipping school or work placement. The current findings were consistent with Kanin (1994). In his study, more than half of the complainants used the false allegation to cover up other behavior. Nine complainants used the complaint to attract attention and three complainants to gain sympathy. Kanin combined both categories and found that 18% of complainants used the false allegation to gain sympathy or attract attention. The categories attention and sympathy are closely related but were kept separate in the current study because there is a noticeable difference between attracting attention and gaining sympathy. In case an allegation is used to attract attention, the valence or the sort of attention that is sought is not relevant. For example, being questioned by the police is a form of attention as well as telling the rape story to a social worker. The attention in itself is the goal. In case an allegation is used to gain sympathy, the complainant tries to improve a personal relationship by filing a false allegation. For example, the complainant wants her father to like her or wants to reconcile with an ex-lover. Five complainants used the false allegation as revenge, to retaliate against a rejecting male. The number of complainants that was motivated by revenge was smaller in the current study than in the study by Kanin. Kanin found that 12 out of 45 complainants were motivated by revenge.

As such, the three motives found by Kanin (1994) were replicated in the current study, but the three motives appeared to be insufficient to explain all the different motives complainants used for filing a false allegation. “Regret” and “Relabeling” were not included in the list of Kanin, but seem to be valid categories. Three complainants filed a false allegation because they regretted a consensual sexual encounter. Two complainants regretted and were ashamed that they had engaged in group sex activities. Two complainants filed a false allegation because they relabeled a consensual sexual encounter as rape. One could argue that the two categories should be combined because some form of regret forms the basis for both. There is, however, a difference between regretting a consensual sexual encounter and relabeling a consensual sexual encounter as rape. The complainants who regretted the consensual encounter tried to alleviate the negative feelings or restore their self-image by filing a false allegation. The complainants who relabeled the consensual sexual encounter deceived the police saying they were raped although they knew they had consented at the time of the sexual encounter and as a result of external pressure had labeled the consensual sexual encounter as rape which resulted in a point of no return.

Four complainants had two motives for filing a false allegation. It appears, therefore, that all the motives listed in the current study are not discrete categories. There is overlap at the category level as well as at the subcategory level. One complainant was motivated by material as well as emotional gain. Three complainants were motivated by two different emotional motives, alibi and revenge, revenge and attention, and regret and attention.

Two unexpected categories had to be added to the proposed list. First, it appeared that not all complainants knew why they had filed a false allegation: 21% of all complainants claimed that they did not know it. The finding that people do not always know what motivates their behavior is not a new finding. Nisbett and Wilson (1977) argued that people are not good at introspection. People, in general, fail to perceive what motivates their actions and, in case they do, that perception is not always an accurate reflection of the reality. Since then, many researchers have replicated the finding that introspection is not always right and that people do not always know what motivates their behavior (Johansson, Hall, Sikstrom, Tarning, & Lind, 2006). Although it was not included in the proposed list, it could have been expected based on the literature. Other researchers, however, who studied motivations of complainants to file a false allegation did not report such a category (Kanin, 1994; McNamara et al., 2012). A second unexpected finding was that information on the motivation to file a false allegation was missing in 17.5% of the cases. In these cases, the police officers either did not ask the complainants what their motivation was, or did ask, but failed to document it. Again, this was not reported by other researchers (Kanin, 1994; McNamara et al., 2012). In our study, we were confronted with a large proportion of missing data. The fact that Kanin and McNamara were not confronted with missing data is a surprising finding in itself.

The current study tried to overcome methodological flaws of other studies in the field of motives for filing false allegations (e.g., small sample size, biased sample, and lack of ecological and construct validity) (Kanin, 1994; McNamara et al., 2012). In research, a validity trade-off often is inevitable. If in a study ecological validity is maximized, another validity, such as internal validity, is often decreased. In the current study, we wanted to maximize ecological validity as much as possible. Thus, all allegations were real false allegations made by complainants to police officers. Stringent criteria were used to firmly establish a ground truth and to prevent that internal validity was compromised. It cannot, though, be excluded that true allegations corrupted the sample of false allegations of rape. In that case, the results retrieved from such a true allegation would be, by definition, not valid.

Another downside of firmly establishing a ground truth was that we probably missed a number of false allegations since unsubstantiated false allegations were excluded. For example, of the 91 allegations classified as false in ViClas 35 had to be excluded because they either did not fulfill our stringent criteria or ground truth could not be established. Besides that, a lot of allegations of rape were never classified as either a true or a false allegation due to evidence problems. Finally, as the case of Gary Dotson illustrated, sometimes a false allegation can be seen as a true allegation and lead to wrongful convictions because the suspects’ denials were not believed and the false complainant was convincing. Because we could not interview the complainants directly and could only study the criminal files, not all motivations might be documented in a reliable way.

Another factor that could have compromised reliability is the fact that the data-gathering process was conducted by one person. Therefore, Cohen’s kappa or any other inter-coder reliability coefficient could not be calculated. Misclassification of motives cannot be excluded either. Especially, the closely related subcategories of sympathy and attention could have been difficult to tease apart, since valence is the only discerning factor. The current findings relied heavily on the work discipline and ethics of the Dutch police officers who conducted the investigations and constructed the files. The large number of “Unknown” motives was caused by the Dutch police officers who failed to ask or document the motive of the complainants as it is instructed by the Dutch police protocol for vice cases. Vice officers could be sanctioned for not following the protocol. The “Unknown” category in the current study is an illustration of the perils associated with conducting archival studies.

In conclusion, the list of Kanin (1994) is valid but insufficient to explain all the different motives of complainants to file a false allegation. A lot of complainants did not know why they had filed a false allegation. In 17% of the cases, we could not report any motive. Complainants are driven by gain, and all complainants were at least, in part, driven by emotional gain. Most salient motives were consistent with the list of Kanin: alibi, attention, and revenge. Other motives for filing a false allegation were sympathy, regret, relabeling, and mental disturbance.

## Footnotes

1 The source of the figures is a personal communication with NU officer Bauke Stelma, who e-mailed that in total 7409 sexually motivated offenses were entered in ViCLAS from 1997 until 2011. Out of the 7409 cases entered in ViCLAS from 1997 until 2011, 2920 were rape cases.
